# Autoantibody Repertoire in APECED Patients Targets Two Distinct Subgroups of Proteins

**DOI:** 10.3389/fimmu.2017.00976

**Published:** 2017-08-16

**Authors:** Dmytro Fishman, Kai Kisand, Christina Hertel, Mike Rothe, Anu Remm, Maire Pihlap, Priit Adler, Jaak Vilo, Aleksandr Peet, Antonella Meloni, Katarina Trebusak Podkrajsek, Tadej Battelino, Øyvind Bruserud, Anette S. B. Wolff, Eystein S. Husebye, Nicolas Kluger, Kai Krohn, Annamari Ranki, Hedi Peterson, Adrian Hayday, Pärt Peterson

**Affiliations:** ^1^Institute of Computer Science, University of Tartu, Tartu, Estonia; ^2^Quretec Ltd., Tartu, Estonia; ^3^Institute of Biomedical and Translational Medicine, University of Tartu, Tartu, Estonia; ^4^ImmunoQure AG, Düsseldorf, Germany; ^5^Children’s Clinic of Tartu University Hospital, Tartu, Estonia; ^6^Pediatric Clinic II, Ospedale Microcitemico, Cagliari, Italy; ^7^Department of Biomedical and Biotechnological Science, University of Cagliari, Cagliari, Italy; ^8^Department of Pediatric Endocrinology, Diabetes and Metabolism, University Children’s Hospital, University Medical Centre Ljubljana, Ljubljana, Slovenia; ^9^Department of Clinical Science, University of Bergen, Bergen, Norway; ^10^Department of Dermatology, Allergology and Venereology, Institute of Clinical Medicine, University of Helsinki, Skin and Allergy Hospital, Helsinki University Central Hospital, Helsinki, Finland; ^11^Peter Gorer Department of Immunobiology, King’s College, Guy’s Hospital, London, United Kingdom

**Keywords:** autoimmune regulator, autoantibodies, immune tolerance, thymus, autoantigen

## Abstract

High titer autoantibodies produced by B lymphocytes are clinically important features of many common autoimmune diseases. APECED patients with deficient autoimmune regulator (AIRE) gene collectively display a broad repertoire of high titer autoantibodies, including some which are pathognomonic for major autoimmune diseases. AIRE deficiency severely reduces thymic expression of gene-products ordinarily restricted to discrete peripheral tissues, and developing T cells reactive to those gene-products are not inactivated during their development. However, the extent of the autoantibody repertoire in APECED and its relation to thymic expression of self-antigens are unclear. We here undertook a broad protein array approach to assess autoantibody repertoire in APECED patients. Our results show that in addition to shared autoantigen reactivities, APECED patients display high inter-individual variation in their autoantigen profiles, which collectively are enriched in evolutionarily conserved, cytosolic and nuclear phosphoproteins. The APECED autoantigens have two major origins; proteins expressed in thymic medullary epithelial cells and proteins expressed in lymphoid cells. These findings support the hypothesis that specific protein properties strongly contribute to the etiology of B cell autoimmunity.

## Introduction

Many severe multifactorial autoimmune diseases are in part defined by pathognomonic antibodies that provide important clinical tests of disease predisposition or status. However, whereas our knowledge of the genetic and cellular factors contributing to multifactorial autoimmune diseases is increasing, we remain largely ignorant of properties of autoantigens and why only selected proteins are targeted in autoimmunity (1). To investigate B cell autoantibody repertoire, we have examined APECED (autoimmune polyendocrinopathy with candidiasis and ectodermal dysplasia) patients (2), defined by rare monogenic defects in the autoimmune regulator (AIRE) gene that drives the expression of tissue-restricted self-antigens in medullary thymic epithelial cells (mTEC) (3–5). T cell progenitors with strong reactivity toward such self-antigens are eliminated or functionally inactivated. Lacking AIRE function, APECED patients predictably accumulate many autoreactive T cells, but in addition they collectively display high titer autoantibodies to multiple self-proteins (2).

The antibodies with the highest reported titers target type I interferons (IFNs) and have become diagnostic or even prognostic markers for APECED (6). Neutralizing type I IFN antibodies mostly appear early in life (7), inhibit IFN-stimulated gene expression (8), and were recently shown to correlate inversely with the onset of type 1 diabetes (T1D) in APECED (9). A second group of APECED-associated autoantibodies targets Th17-associated cytokines, IL17A, IL17F, and IL22, emerges early in the disease course (7), and is associated with chronic mucocutaneous candidiasis (10, 11), another defining feature of APECED. Interestingly, our in-depth studies of anti-cytokine antibodies harbored by APECED patients showed that the functionally rearranged immunoglobulin gene sequences were heavily mutated, encoding antibodies of extremely high affinity that we hypothesized to result from B cell dysregulation in germinal center reactions (9). Such etiology can be contrasted with the amplification and maturation of natural, polyreactive autoantibodies that are normally of relatively low affinity, which has been proposed to underpin sporadic autoreactivity in otherwise healthy individuals (12).

There is a prominent set of APECED-associated autoantibodies that are shared with multifactorial autoimmune diseases, such as those directed against glutamic acid decarboxylase (GAD)1 and GAD2 in T1D (13), or against CYP21A2 in Addison’s disease (14). Therefore, the comprehensive analysis of autoantibodies in APECED may offer improved insight into the etiology of such diseases. These autoantibodies have often been identified by candidate or cDNA expression library screenings. However, proteome arrays provide unprecedentedly broad coverage to identify novel target proteins, such as melanoma MAGEB antigens and testis-specific PDILT, which were recently identified as autoantigens in APECED (15).

To gain further insight, we searched common features of autoantigenic proteins by a systematic analysis of a protein microarray platform, which allows large-scale, unbiased screening of autoantibody reactivities. Herein, we report a broad spectrum of autoantigens that is collectively targeted by 82 APECED patients. This APECED “autoimmunome” is not a random collection of proteins but comprises two sub-groups, those whose expression is most likely AIRE-regulated in the thymus and those enriched in lymphoid tissue. Moreover, these autoantigens are enriched in evolutionarily conserved cytosolic and nuclear proteins with high propensity for post-translational modifications. Reactivity to an increased number of such autoantigens was a stronger correlate of clinical symptoms than was either patient age or time since disease onset.

## Materials and Methods

### Patient Samples

Altogether 100 sera samples from 82 individual patients were profiled in Protoarray, as described (9). For longitudinal analysis, we collected 2 samples from 11 patients; 3 samples from 2 patients; and 4 samples from 1 APECED patient. Only one serum sample (with maximal number of positive hits) per patient was used in all analyses, except in longitudinal analysis where multiple samples taken at different time points were available from 14 patients. The patients were from Finland, Italy, Norway, Slovenia, and Estonia and diagnosed according to mutational analysis of the AIRE gene and by the presence of autoantibodies to type I IFNs. The mean age of the patients was 31 years. The main characteristics of the patients are given in Table S1 in Supplementary Material. The control group consisted of 20 individuals (12 healthy volunteers and 8 healthy first-degree relatives of the Italian APECED patient cohort). The study was carried out in accordance with the recommendations of local ethics committees (Finland: HUS Medical ERB, 8/13/03/01/2009; Slovenia: National Medical Ethics Committee number 22/09/09 and 28/02/13; Italy: Ethics Committee Prot. PG/2015/20440; Norway: Research Ethics Committee of Western Norway, health registry number 047.96, bio-bank number 2013-1504, project number 2012/1850; Estonia: Research Ethics Committee of the University of Tartu, 235/M-23) with written informed consent from all subjects, as described earlier (9). All subjects gave written informed consent in accordance with the Declaration of Helsinki.

### Protein Array Screening

The autoantibody screening was performed by ISO9001 certified Custom ProtoArray Service Lab at Invitrogen (Thermo Fisher Scientific). Briefly, the protein arrays (ProtoArray v5.1) were probed as described in Invitrogen’s protocol for Immune Response BioMarker Profiling using detection reagent (Alexa Fluor 647 Goat Anti-Human IgG A21445, Invitrogen) and blocking buffer (Blocking Buffer Kit PA055, Invitrogen). Arrays were scanned using a GenePix 4000B fluorescent scanner, and the data were acquired with GenePix^®^ Pro software. The arrays were probed with sera at a dilution of 1:500.

### Data Cleaning, Normalization, and Print Contamination

Previous studies involving protein microarrays (16) have shown that raw intensity values should not be compared directly because of technical, physical, chemical, and individual variability, occurring mostly at the production stage. In order to preserve true biological signal and concurrently eliminate potential technical noise, we applied a robust linear model (RLM) approach (17), using *rlm* function in R. RLM is considered to be the state of the art normalization technique for protein microarray data (18) and is applied on control probes that are assumed to have constant positive signal across all arrays. We used human-IgG and V5 control series for normalization as reported (17). Background subtracted signal was log-transformed prior to applying RLM to approximate for normal distribution (Figure S1 in Supplementary Material). To address printing contamination, we excluded all autoantibody targets that showed high correlation (>0.6) with previously reported autoantigens. In addition, we identified and eliminated highly correlated proteins located in the neighboring wells on the array. In total, we identified and removed 31 false positives, some of which were prevalently positive across many samples (Figure S2 in Supplementary Material). The normalized signal was standardized using mean and SD of healthy samples (including healthy heterozygous relatives). Proteins, with standardized signal (*z*-score) above 3 in three or more patients were regarded as autoantigens. The full list of the positive proteins is provided in Table S2 in Supplementary Material.

### Differential Data Analysis and Clustering of Autoantibody Reactions

In order to identify the proteins correlating with various clinical manifestations, we used moderated t-statistics implemented in *ebayes* function from *limma* R package (19) on normalized intensities from all 9,000 proteins. Obtained *p*-values were corrected using false discovery rate correction method and top significant proteins (corrected *p*-value <0.05) were extracted for each manifestation resulting in six significant associations. Unsupervised hierarchical clustering using *pheatmap* package in R, which internally uses *hclust* function for clustering rows and columns of matrix. Linear regression analysis of dependency between number of positive hits and number of manifestation was analyzed with *lm* method in R.

### Variability of APECED Autoantigen Profile over Time

We extracted data for 14 patients with multiple samples and formed all possible pairs of samples that belong to the same patients and divided these pairs into two broad categories: pairs of samples that were obtained >10 years apart (eight samples, on average 24.4 years apart) and samples that were obtained <10 years apart (13 samples, on average 1.5 years apart). Reactive proteins were compared between samples of the same patient and the percentage of the proteins that were specific to early, late, or shared between samples for both of the categories was computed. As expected, samples taken close in time had more reactive proteins in common, then samples obtained more than 10 years apart. Samples taken more than 10 years apart show higher percentage of late sample specific proteins, which might mean that with time repertoire of reactive proteins gets larger.

### Correlation with APECED Mutations

We divided the patients into three groups based on their mutations: (1) homozygous for p.R139X, (2) homozygous for p.R257X, and (3) homozygous p.L323fsX373 or compound heterozygotes of either p.L323fsX373 or missense mutations. We used pairwise comparisons of Tukey and Kramer (Nemenyi) test with Tukey-Dist approximation for independent samples.

### The Enrichment Analyses for Protein Characteristics

To assess intrinsic features of positive reactivities, we used data from the following public databases: Human Protein Atlas (20), Compartments DB (21), dbPTM (22), UniProt Knowledgebase (23), Ensembl (24), OrthoDB8 (25), and SUPERFAMILY (26). We used *gconvert* function from *gProfileR* package in R (27) to convert names of genes and proteins into Ensembl gene identifiers (ENSG). Size of the overlap between our group of positive proteins and every set extracted from each database was estimated. Using hypergeometric test for each overlap, we computed probability values to estimate how likely it was to observe this overlap or larger by random chance. We used false discovery rate procedure to adjust obtained *p*-values and significance threshold of 0.05 to reject a null-hypothesis. To double check our findings, we used g:Profiler web-tool (27) by submitting our list of positive proteins and running unordered query with ProtoArray content as a background to account for any design bias.

### Single Nucleotide Polymorphism (SNP) and Evolutionary Conservation Analysis

For SNP analysis, we retrieved all SNPs associated with each gene on our platform that was found in Ensembl database (7,284 genes). To retrieve this information, we used R command useMart and the following parameters: useMart(“ENSEMBL_MART_ENSEMBL”, dataset = ‘hsapiens_gene_ensembl’, host = “www.ensembl.org”). We then calculated the number of SNPs within boundaries of the gene and SNPs that are located at the exon regions of the gene. We normalized these values by associated gene length (distance between two furthest associated SNPs), by gene length, and by accumulative length exon regions of each gene, respectively. To compare positive proteins with the rest of platform in terms SNP counts, we first sampled 10,000 random groups of genes of the same size as our group of reactive proteins. For each group, we computed mean counts of two categories. For evolutionary conservation analysis, the data were obtained from ftp://cegg.unige.ch/OrthoDB8/Eukaryotes/. We downloaded four pairs of genes and annotations for all_species, mammalia, metazoa, and vertebrata. In all four gene files, we filtered out genes not related to *Homo sapiens* and converted Ensemble protein IDs into gene IDs using biodbnet (http://biodbnet.abcc.ncifcrf.gov/db/db2db.php) software. We calculated an average evolution rate for our group of reactive proteins (for all genes that had evolutionary rate recorded in DB). Then we sampled 10,000 random groups of the same size as our reactive group and calculated an average evolutionary rate.

### Luciferase Immunoprecipitation (LIPS) Analysis

Luciferase immunoprecipitation assay was performed as reported (28, 29). Autoantigen cDNAs were cloned into modified pNanoluc vector (Promega, Madison, WI, USA) downstream of naturally secreted NanoLuc luciferase (Nluc). HEK293 cells were transfected with the plasmid constructs and cell culture medium containing Nluc-fusion proteins was collected after 48 h. Serum samples were first incubated with the fusion protein solutions overnight, then Protein G agarose beads were added and incubated at room temperature for 1 h in 96-well microfilter plates (Merck Millipore, Billerica, MA, USA) to capture antibodies and immune complexes to the beads. After the washing to remove unbound fusion proteins, a luciferase substrate (furimazine) was added to reaction, and luminescence intensity (LU) was measured in Victor X Multilabel Plate Reader (PerkinElmer Life Sciences, Waltham, MA, USA). Results were expressed as relative units LU sample/average LU of healthy control samples. The positive/negative discrimination level was set to the mean plus three SDs of the healthy control samples.

## Results

### Protoarray Screening of APECED Sera

The basis for our unbiased study of APECED autoreactivities was a protein microarray (Protoarray) of approximately 9,000 proteins printed to a single glass slide. Against this array, we screened 100 sera from 82 patients of Finnish, Norwegian, Slovenian, Sardinian, and Estonian origins, together with control sera obtained from healthy heterozygous relatives (*n* = 8) and unrelated healthy controls (n = 12) across the same age range.

The arrays were normalized by RLM (17) based on control protein intensities with further standardization and limitation of false-positives (described in Materials and Methods). The positive proteins were defined as signals with a z-score (the number of standard deviations SDs from the mean of combined controls) of greater than 3 (which is more stringent than the common practice of calling hits at z > 2). Although APECED patients altogether displayed significantly different set of autoreactivities in comparison to controls, the reactivity profiles contained multiple patient-specific reactivities in addition to shared autoantibodies (9). Therefore, to intensify the likely biological and clinical significance of hits, we considered a protein to be an autoantigen only when it was recognized by ≥3 individual patient samples (see Materials and Methods). When this additional stringency was applied, proteins encoded by 963 unique Refseq genes qualified as autoantigens.

### Autoantigen Repertoire Contains Shared and Individual Reactivities

**Figure 1:**
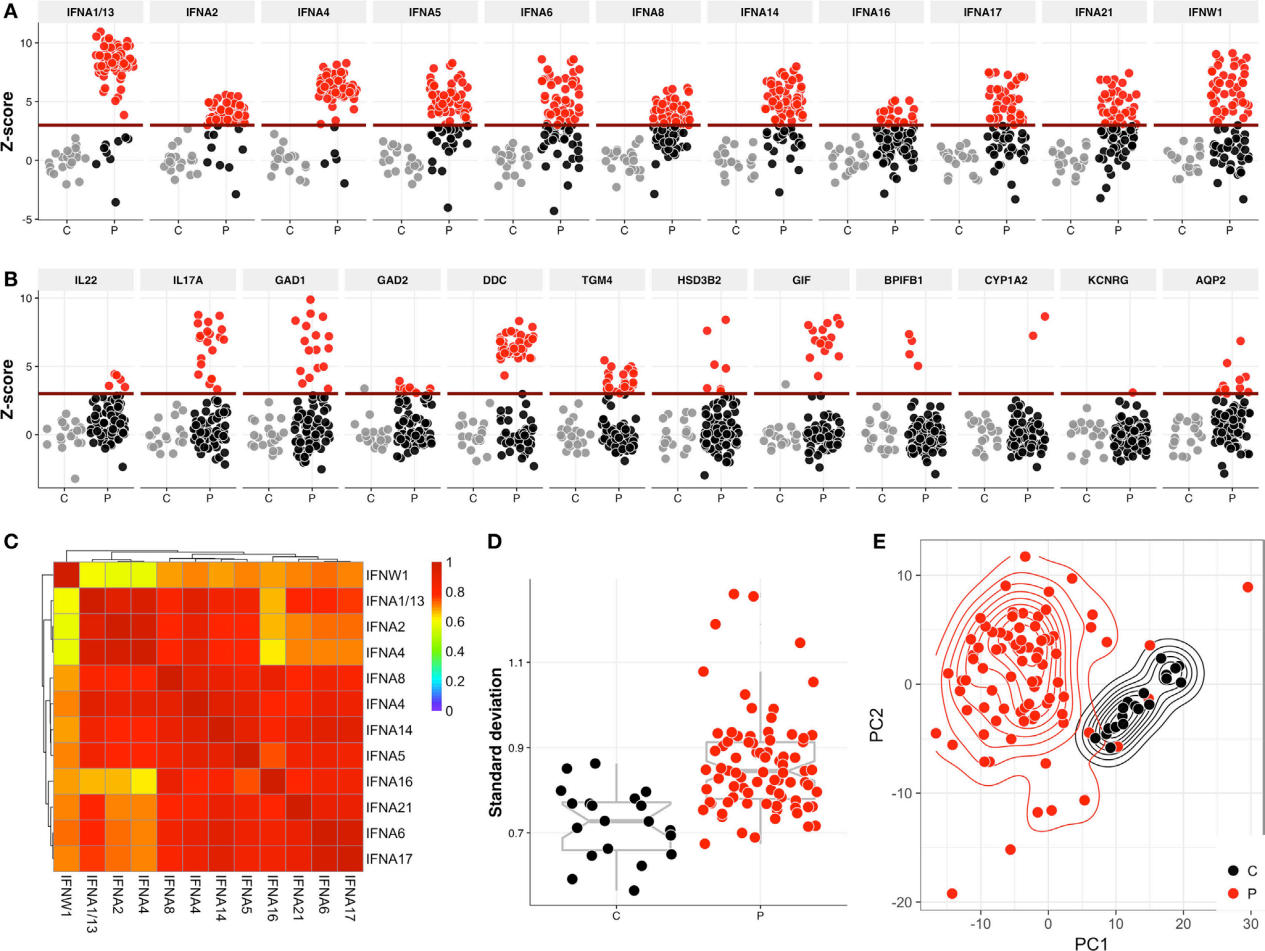
Protoarray reactivities to type I interferons (IFNs) and to known autoantigens. The reactivities to **(A)** type I IFNs and **(B)** known APECED autoantigens. The Protoarray signals are expressed as *z*-scores representing the number of SDs from the mean of combined control samples. Positive-negative discrimination level (dark red line) was set at *z* = 3. Red circles represent the samples with *z* > 3, gray circles are control samples. **(C)** Heatmap of correlation coefficients of type I IFN reactivities between each other. **(D)** Variation analysis of controls and patients—each dot on this figure represents a SD within sample over all Protoarray signals. Red circles represent patients (P) and black controls (C). Variance in patient samples are significantly higher (*p* = 2.598e−07) in comparison to control samples. **(E)** Principal component analysis plot of controls and patients. The first two principle components explain 35.8 and 11.5% of total variance, respectively. First 50 most common autoantibody reactivities were used in order to build this plot.

As expected, multiple sera displayed strong reactivities toward approximately 20 proteins that were previously reported as APECED autoantigens, including type I IFNs (Figure 1A), Th17 cytokines, GAD1, GAD2, DDC, TGM4, GIF, HSD3B2, BPIFB1, TSGA10, CYP1A2, KCNRG, and AQP2 (Figure 1B). Moreover, most such reactivities were also detected by LIPS assay (Figure S1 in Supplementary Material). It should be noted that apart of HSD3B2, the set of proteins on Protoarray did not include the steroidogenic enzymes CYP21A2, CYP17A1, and CYP11A1, which have been demonstrated as autoantigens in APECED.

Among shared reactivities, the highest titers were shown by interferon-α (IFNA)-specific antibodies, for which we identified some strong correlations (coefficients from 0.41 to 0.98), the highest being between IFNA2 and IFNA4 (correlation ~0.98), and IFNA6 and IFNA17 (correlation ~0.93) (Figure 1C). These correlations could not be explained by the levels of primary sequence similarity or by close phylogenetic relationships (Figure S2 in Supplementary Material), and are instead likely to reflect shared conformational epitopes in IFNA proteins. Likewise, there was commonly a strong correlation (coefficient from 0.41 to 0.62) of IFNA reactivity with reactivity toward IFN-ω (IFNW) (Figure 1C).

In addition to shared reactivities, we found high inter-individual variation in the number and identity of autoantigens (Figure 1D). To analyze a covariance of sample groups, we performed a principal component analysis, which showed that the relatively homogenous group of combined healthy relatives and control samples readily segregated from the patient samples that displayed a large intra-group variability (Figure 1E).

### Multiple Specificities Include Cancer–Testis and Testis-Specific Autoantigens

**Figure 2:**
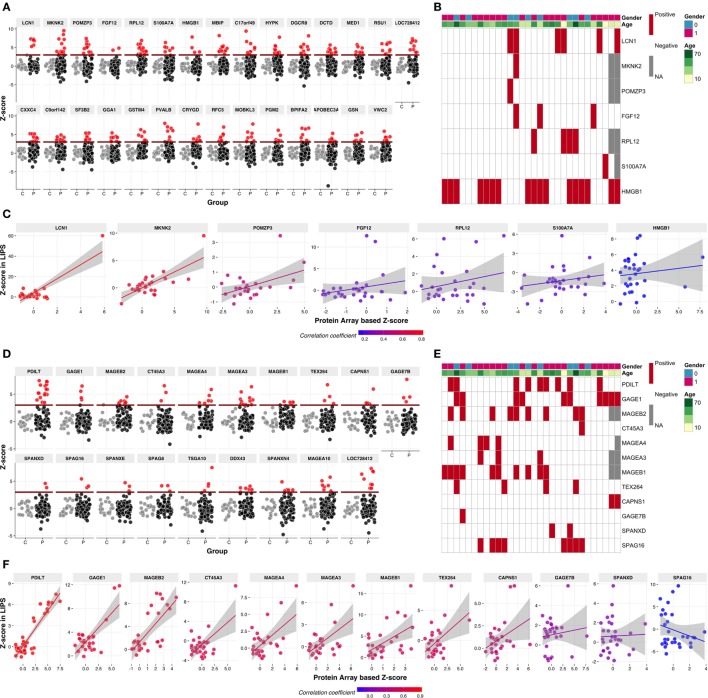
Autoantibody reactivities to selected novel autoantigens. **(A)** The Protoarray signals are expressed as *z*-scores representing the number of SDs from the mean of combined control samples. Positive–negative discrimination level (dark red line) was set at *z* = 3. Red circles represent the samples with *z* > 3, gray circles are control samples. **(B)** Seven out of the 29 novel autoantigens were tested in luciferase immunoprecipitation (LIPS) using a subset of 30 Finnish APECED patients’ samples. Red color in the cells of the heatmap represents positive reactivity (*z* > 3), gray cells indicate missing values and white color stands for missing autoantibodies. Rows on top indicate gender and age. **(C)** Correlation analysis of Protoarray and LIPS results on the autoantigens in panel **(B)**. **(D)** *z*-Scores to 19 cancer–testis antigens (CT-As) in APECED patients according to Protoarray results coded as in panel **(A)**. **(E)** Twelve of identified CT-As were retested using LIPS assay in a subset of 30 Finnish APECED patients. Color code as in panel **(B)**. **(F)** Correlation analysis of Protoarray and LIPS results of CT-As in panel **(E)**. Color of dots and fitted linear trends in panels **(C,F)** represent the value of correlation coefficient, higher coefficient values are encoded as red color and low or negative correlation as blue.

Among the strong, “rare” specificities, there was a number of potential autoantigens depicted in Figure 2A. Of these, LCN1 was reported as an autoantigen in Sjögren syndrome patients (30), and HMGB1 was reported to be associated with the production of anti-DNA autoantibodies in systemic lupus erythematosus (31). To extend the Protoarray results, we used LIPS to assay reactivity toward eight of these proteins (LCN1, MKNK2, POMZP3, BAALC, FGF12, HMGB1, RPL12, and S100A7A) of sera from a subgroup of 30 Finnish APECED patients (Figure 2B). The first three showed particularly strong positive correlations between Protoarray and LIPS results (Figure 2C), but given that the reactivities were often rare and that the patient groups tested in the two assays did not completely overlap, it was not surprising that correlations were not always statistically significant. Of note, however, this did not reflect a general tendency of the Protoarray to reveal false positives, since some sera showed much stronger LIPS reactivity to defined targets (e.g., RPL12, FGF12, HMGB1; Figure 2C). Indeed, this may reflect the potential of the Protoarray to underestimate reactivities of conformation-specific autoantibodies, because of the denatured states of many of the arrayed proteins and protein fragments.

Interestingly, we noted reactivities toward ~20 so-called “cancer-testis autoantigens” (CT-As) (Figure 2D). CT-As can be expressed in a wide variety of malignant tumors, where they are known to be immunogenic, but their expression in normal tissue is mostly restricted to germ cells in testis, fetal ovary, and placenta (32). The extent of reactivity revealed by our screen far exceeded the previous report of shared APECED reactivity to CT-As PDILT and MAGEB2 (Figure 2D) in a large-scale screening of another APECED cohort (15). Rather, we found reactivities toward several proteins from the MAGE-A—MAGE-B family that are expressed in melanoma and other tumor types but which in normal tissues are expressed only in the testis; reactivities toward GAGE1 and GAGE7B, which are members of another X-chromosome linked, CT-A family; and reactivities toward SPAG8 and SPAG16 (Figure 2D). The latter two in particular highlight the cross-over of APECED serum autoreactivities with other pathophysiologies, in that SPAG8 was initially identified as a sperm-associated antigen target of serum of an infertile woman (33), while SPAG16 is expressed in sperm and in reactive astrocytes of lesions in multiple sclerosis patients in whom it has been identified as an autoantibody target (34). Of note, both males and females showed autoreactivities toward several sperm-specific proteins (Figure 2E). Protoarray reactivities toward 11 testis specific and CT-antigens tested showed good overall correlation with LIPS (Figures 2E,F).

### Autoantibody Correlations with Clinical Manifestations

**Figure 3:**
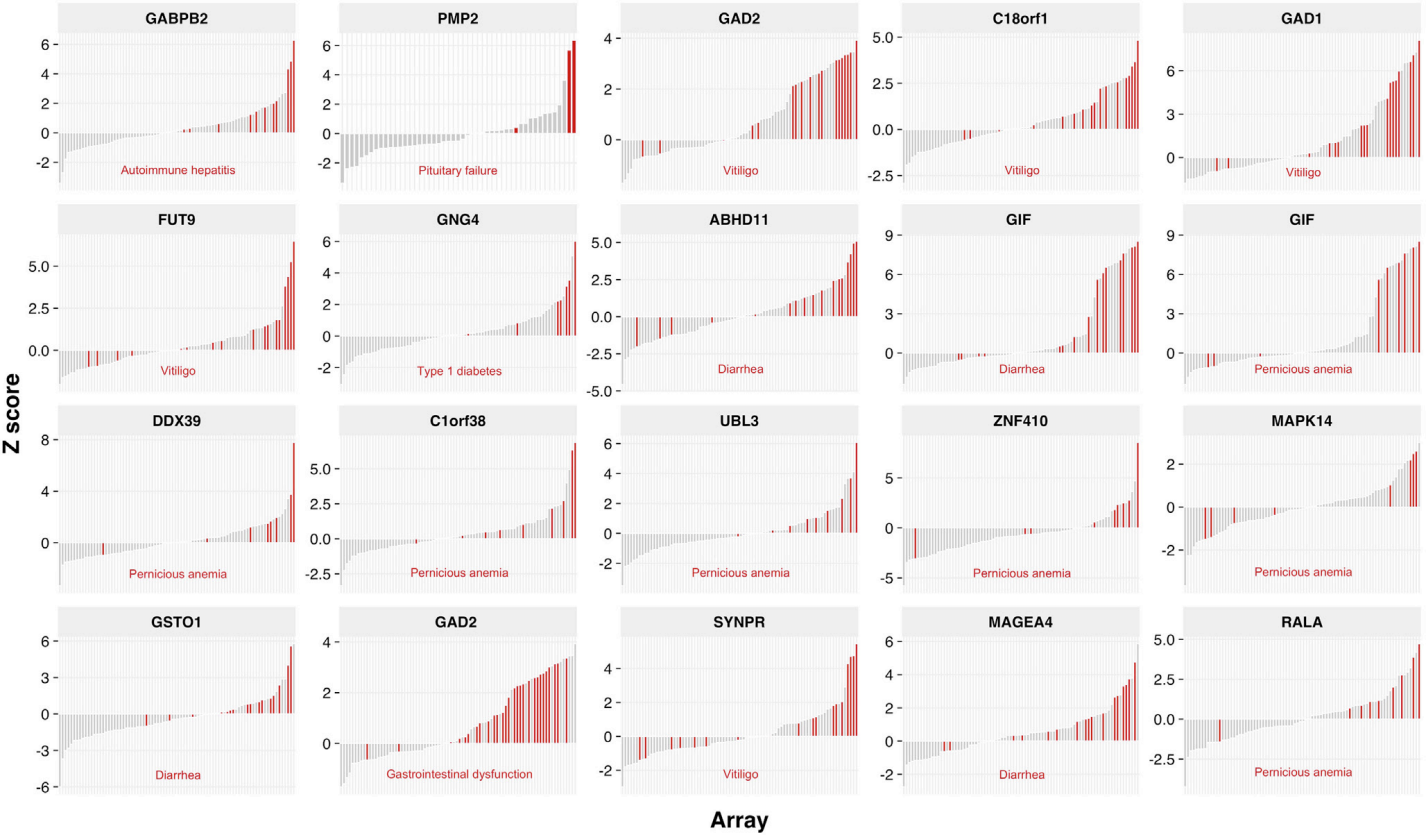
Autoantibody reactivities associated with APECED patients clinical and phenotypic features. Association of Protoarray *z*-scores with corresponding clinical manifestations. The height of the bar depicts *z*-score of the sample, red bars represent samples positive for a given manifestation (indicated below each plot). All other bars in gray represent autoantibody levels in patient samples without the certain clinical manifestation. Only statistically significant associations are shown (*p* < 0.05) and adjusted across 900 most reactive proteins on Protoarray using moderated contrast *t*-test.

We next analyzed whether immunoreactivities to certain autoantigens correlated with disease components of individual APECED patients (Figure 3). Pernicious anemia correlated with seven reactivities, including gastrointestinal factor GIF that is an established autoantigen in pernicious anemia patients (35); five specificities, including GAD1 and GAD2, correlated with vitiligo; and autoantibodies to GABPB2 correlated with autoimmune hepatitis in APECED patients. By contrast, several associations described previously among small-scale studies, e.g., DDC with AIH and vitiligo (36, 37), were not revealed. In addition, we confirmed that reactivity toward prostate specific antigen, TGM4, was almost restricted to post-pubertal males (Figure S3 in Supplementary Material) (38).

**Figure 4:**
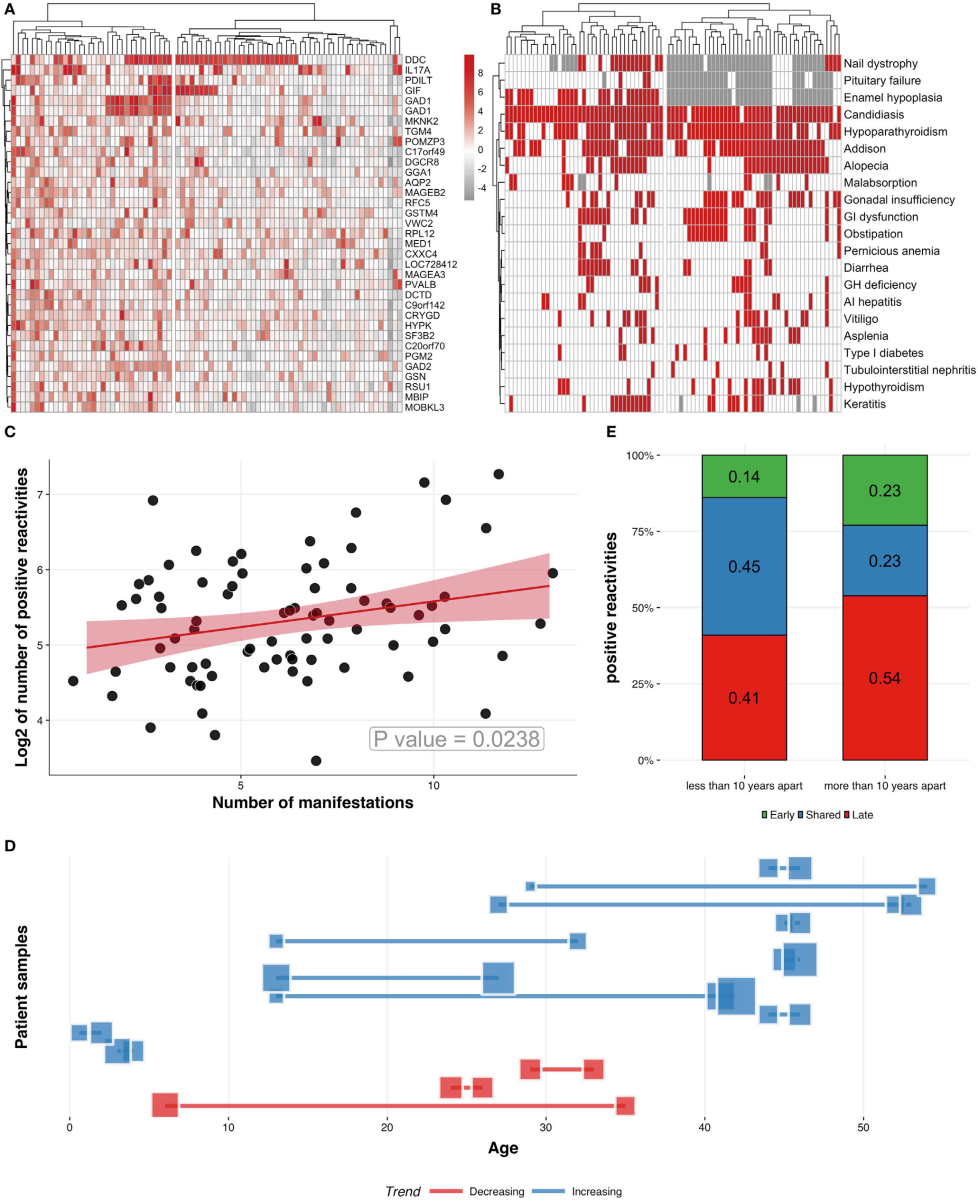
Characteristics of APECED patients and Protoarray reactivities. **(A)** Clustering patients and *z*-scores for the 50 most commonly recognized autoantigens using hierarchical clustering algorithm. **(B)** Clustering patients (columns) according to their clinical manifestations (rows). Red squares represent the presence of a given manifestation, while white and gray show absence of manifestation and missing information, respectively. **(C)** Positive correlation between the number of manifestations and the number of autoantigens (*z* > 3, logarithmized), correlation coefficient ~0.29. **(D)** Analysis of longitudinal serum samples taken at different time points. Blue squares and connecting lines (*n* = 11) correspond to samples with increased number of autoantigens and red squares and connecting lines (*n* = 3) correspond to samples with decreased or unchanged number of autoantigens. Each line and connected squares correspond to one APECED patient with samples collected at different time points. The locations of the squares on *y*-scale correspond to the age of the patient when the samples were collected. The sizes of the blue and red squares correspond to the number of positive autoantigens in corresponding samples. **(E)** The change of autoantigen profiles in samples collected longitudinally. The two columns show the autoantibody reactivities in patient samples taken less than 10 or more than 10 years apart. The autoantigens were compared between the samples of the same patient and divided into three categories according to their specificity: (i) specific to early sample, (ii) shared in early and late sample, and (iii) specific to late sample. The numbers in columns indicate the proportions of each category. Samples taken more than 10 years apart show higher percentage of autoantigens in the late samples, indicating that the autoantibody repertoire gets broader with time.

To test whether the autoantibody reactivities might segregate APECED patients into subgroups, we performed unsupervised hierarchical clustering of patients and the 50 most-reactive autoantigens, from which we excluded type I IFNs. This analysis clearly revealed two patient sub-groups with overall low and high reactivity repertoires, respectively (Figure 4A). An overlay of this heatmap with clinical data showed that the number of autoantigens targeted was positively correlated with the number of clinical manifestations, as confirmed by linear regression (Figures 4B,C).

As the number of disease components in APECED patients is known to increase over time, we tested whether the number of autoantibody targets in each patient is likewise related to the age of onset of the first clinical manifestation. However, the trend toward correlation between the number of diseases and either age or time since diagnosis was not significant (Figure S4 in Supplementary Material), arguing that neither can independently explain the accumulation of multiple clinical entities in APECED. Instead, these findings add further weight to the conclusion that the complexity of the autoantibody response is a major underlying factor for the expansion of clinical profile in APECED.

Previous studies have shown changes in autoantibody profiles over time. To understand the dynamics of autoantibody repertoire, we studied 14 APECED patients whose sera were collected at least twice in different time points and, thus, analyzed longitudinally by Protoarray. In most patients (*n* = 11), the number of proteins recognized by autoantibodies increased with time (Figure 4D), whereas in only three patients the number of reactivities decreased or remained almost unchanged. We next studied whether the target antigen profile in APECED patients broadens over time. For this, we compared the samples taken less than 10 years apart with the samples spanning more than 10 years in three separate categories: (i) target specific to early sample, (ii) target shared in early and late sample, and (iii) target specific to late sample. Expectedly, the proportion of targets specific to late sample was larger indicating that the samples taken closer in time shared more reactivity to autoantigens than samples obtained more than 10 years apart (Figure 4E).

### Nature of APECED Autoantigens

**Figure 5:**
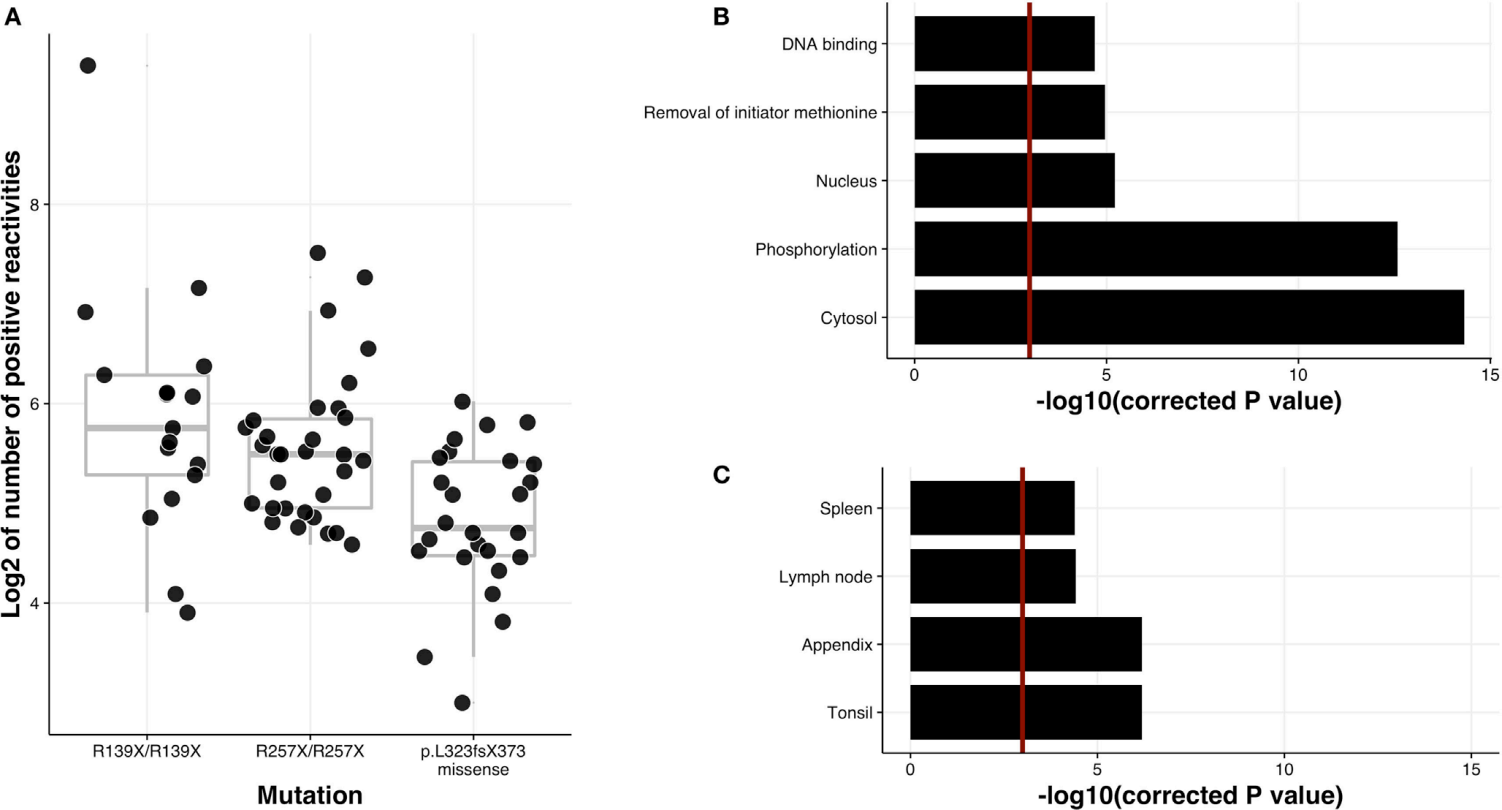
Autoantibody correlation with APECED mutations and enrichment analysis for protein characteristics. **(A)** Correlation of the number of autoantibody targets with APECED mutations using pairwise comparisons with Tukey and Kramer (Nemenyi) test and Tukey-Dist approximation for independent samples. The first and the second mutation types are significantly different from the third type in terms of log2 of positive hits with *p*-values 0.0016 and 0.0072, respectively. **(B)** Association of autoantigens with different protein characteristics. Length of each bar is directly proportional to the significance of the term, calculated with hypergeometric test. False discovery rate was used to correct for multiple testing. Red line is drawn at 0.05 significance level. **(C)** Association of positive reactivities with tissue-specific gene groups from Human Protein Atlas. Hypergeometric test was used to compute displayed *p*-values and false discovery rate was used to correct for multiple testing.

Of the many AIRE truncating mutations identified in APECED patients, the most prevalent are p.R257X creating a premature stop codon in exon 6, and a 13bp deletion, p.L323fsX373, changing the reading frame and causing a premature stop codon in exon 8. Although both mutations are present in APECED patients with various ethnic backgrounds, the R257X is commonly found among Finnish (83% of APECED alleles), Norwegian and Slovenian patients. By contrast, the earliest AIRE truncation occurs in Sardinian patients who share a p.R139X mutation that introduces a premature stop codon prior to the SAND domain (Sp100, AIRE-1, NucP41/75, DEAF-1), an ~80 residue region common to many chromatin-regulating factors. When we divided the patients into three groups based on these genetic etiologies [(1) homozygous for p.R139, (2) homozygous for p.R257X, and (3) homozygous p.L323fsX373, or compound heterozygotes of either p.L323fsX373 or missense mutations], we found patients in Groups 1 and 2 to react to a wider spectrum of autoantigens (Figure 5A), thereby correlating autoantibody reactivities with the p.R139X and p.R257X truncation mutations.

Intrinsic features of proteins that might promote their autoantigenicity are not known. Indeed, the available information on the repertoire of autoantibody target proteins is fragmented, and based on this, few if any overt similarities have emerged among proteins identified as autoantigens. Given the scale of the analysis described here, we systematically analyzed our list of autoantibody target proteins for any parameters that might be enriched relative to proteins that were not targets (Figure 5B; Table S2 in Supplementary Material).

We first studied the subcellular localizations of APECED autoantigens by employing two different approaches: (i) the Compartments resource that integrates sequence-based and manually curated subcellular localization information from PSORT and YLoc databases and (ii) g:Profiler that searches for a cellular component as one of the Gene Ontology categories (27). The majority of autoantigens were significantly associated with intracellular location (adj. *p*-values 2.62e−07 and 7.20e−03 for Compartments and g:Profiler analysis, respectively), and in particular with either a cytosolic location (adj. *p*-values 6.04e−07 and 3.01e−05 for Compartments and g:Profiler analysis, respectively) or, to a lesser extent, a nuclear location (*p* = 0.0054) (Table S2 in Supplementary Material).

Posttranslational modifications have been proposed to contribute to autoimmune responses (39). Indeed, when we compared our database with dbPTM datasets (22) comprising an integrated resource for protein posttranslational modifications, we found that the autoantigens were strongly enriched in proteins with histidine/serine/threonine phosphorylation (adj. *p* = 3.34e−06 for HST). The association was significant even when threonine and serine phosphorylation were compared separately (adj. *p*-value 4.22e−06 for threonine only and 8.43e−04 for serine only). However, tyrosine phosphorylation alone was not enriched and adding tyrosine phosphorylation as a category to the accumulated list of phosphorylated proteins decreased the significance level. We searched for the enrichment of specific protein features in UniProtKB and Superfamily databases comprising a collection of protein function information. This analysis surprisingly revealed that autoantigens were enriched for proteins from which the initiator methionine is processed by methionine aminopeptidases (adj. *p* = 0.0070) and representation of proteins with DNA binding potential (adj. *p* = 0.0091) (Supplementary Table S2 in Supplementary Material).

**Figure 6:**
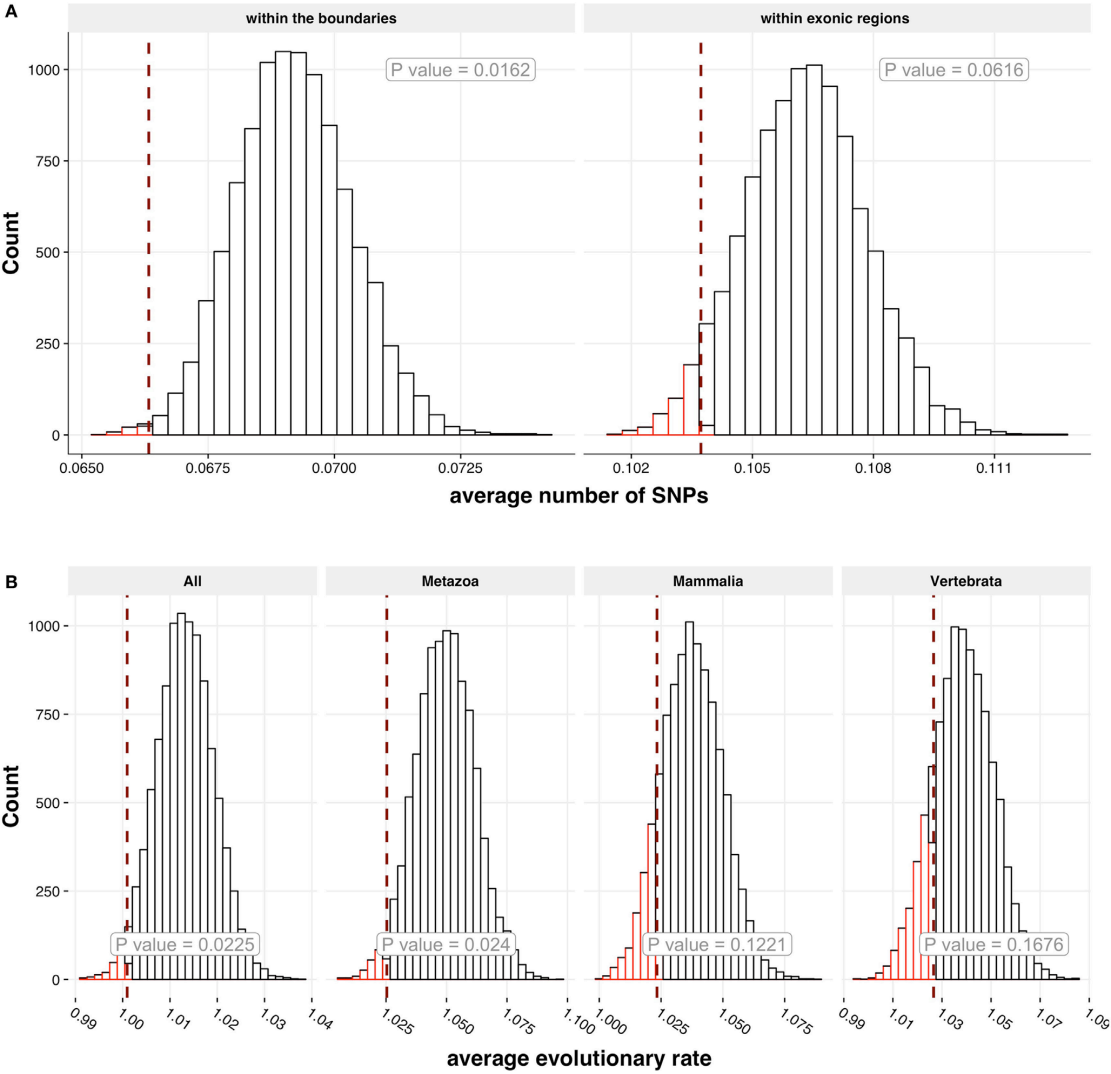
Genetic polymorphisms and evolutionary conservation of APECED autoantigens. **(A)** Association of autoantigens with single nucleotide polymorphisms (SNPs). Two SNP categories were analyzed: the number of SNPs (i) within the boundaries of a gene (left histogram) and (ii) within exon regions of a gene (right histogram). For both histograms 10,000 randomly sampled groups of Protoarray proteins (genes) were generated with the size equal to the size of positive autoantigen group. On the *x*-axis the average number of SNPs is shown. The red dashed line shows the number of SNPs in positive autoantigen group. The *p*-value for each category of SNPs has been calculated in order to estimate the significance of a difference between randomly sampled groups and positive group. The autoantigen group has lower mean normalized SNP count, then it would be expected on average for the genes encoding proteins on Protoarray. **(B)** Evolutionary conservation rate. The four histograms that show distribution of average evolution rates for 10,000 randomly sampled groups of Protoarray genes were generated with the size equal to the size of positive autoantigen group. Each histogram represents a comparison of positive autoantigen group with ortholog genes in different evolutionary categories (*all, metazoa, mammalia*, or *vertebrata* species). On the *x*-axis the average evolutionary rate is shown. The red dashed line represents an average evolutionary rate of positive autoantigen group. Computed *p*-values indicate that autoantigens are on average more conserved in categories *all* and *metazoa* than the rest of the genes encoding proteins on Protoarray.

The genes encoding autoantigens have been suggested to be enriched for SNPs (40). However, when we studied SNP data from Ensembl.org, the genes encoding APECED autoantigens displayed fewer SNPs in their gene regions (*p* = 0.0126) as well as in exons (*p* = 0.0592) (Figure 6A). To study how evolutionary conserved the APECED autoantigen gene families (41) are, we used OrthoDB8 database to retrieve the calculated evolutionary rates of the autoantigen genes (as human counterparts) against ortholog genes in four different taxonomic levels (all species, metazoan, vertebrata, and mammalian species). We found that the genes encoding APECED autoantigens showed significant association with the two conserved taxonomic levels (all species, *p* = 0.0225; metazoan species, *p* = 0.024; and a similar trend in mammalian and vertebrata species categories) (Figure 6B) indicating an evolutionary conserved nature of APECED autoantigens. Thus, in comparison to all genes in the genome, APECED autoantigen genes have less genetic polymorphisms and are more conserved in evolution.

### Autoantigens Segregate Based on mTEC Expression and AIRE Dependence

The key role of AIRE is considered to be the establishment of tolerance toward proteins restricted to discrete peripheral tissues, the so-called tissue-restricted (TR) antigens. However, unexpectedly, we did not find the APECED autoimmunome to be appreciably enriched in TR antigens. In fact, when each tissue was analyzed separately using the Human Protein Atlas database (20), the APECED autoantigens showed the strongest significant correlation with gene expression in lymphoid tissues, specifically tonsils, spleen, appendix, and lymph nodes (Figure 5C; Table S2 in Supplementary Material).

**Figure 7:**
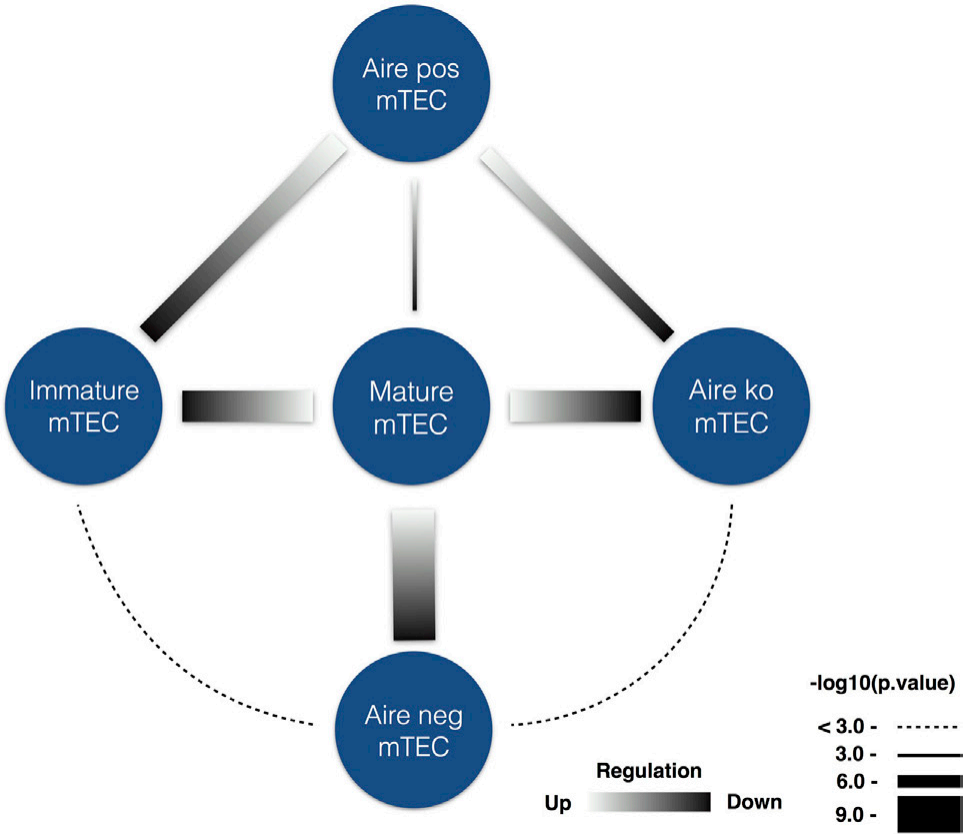
Medullary thymic epithelial cells (mTEC) specific expression of tissue-restricted (TR) APECED autoantigens. TR APECED autoantigens but not the non-tissue-restricted (NTR) autoantigens were upregulated in mature Aire-expressing mTECs. Datalists [generated by Sansom et al. (42)] of differentially expressed genes in mTEC subsets were compared to the list of APECED autoantigens. Strong enrichment of TR autoantigens was found among differentially upregulated genes in mature mTEC subpopulation in comparison to (i) immature mTECs, (ii) Aire-negative mTECs, and (iii) Aire-knockout mTEC populations. Blue circles mark the corresponding cell populations, the gradient in connecting lines indicate whether the genes in comparison were up- or downregulated, and the width of the lines represents the logarithmized *p*-value. No significant overlap was found among NTR autoantigens differentially expressed in any of the studied thymic cell comparisons.

These findings notwithstanding, given that AIRE is the driving cause of APECED, we hypothesized that the autoantibody repertoire might target two components: TR antigens and non-tissue-restricted (NTR) antigens enriched in AIRE-independent lymphoid tissue proteins. To test this hypothesis, we divided the genes encoding the autoantigens according to their TR or NTR expression pattern (Table S2 in Supplementary Material). We then cross-referenced mouse databases on the expression of genes by mature mTECs and to its subsets, including those where thymic AIRE expression is limited or lacking by knockout approach (42) (note the necessity to use mouse databases reflected by the lack of comprehensive human thymus gene expression data). Having converted human gene accession numbers to mouse counterparts, we found a very strong overlap of genes encoding APECED TR autoantigens with genes more highly expressed in mature mTEC compared to two Aire-negative subsets: immature mTEC (*p* = 0.00022) and Aire-negative mTEC subpopulation (*p* = 0.00016) (Figure 7). The genes downregulated in Aire-deficient mTEC cells were likewise enriched in genes encoding APECED TR autoantigens (*p* = 0.00022). By contrast, the NTR group showed no such correlations. Furthermore, the NTR group was associated even more strongly with intracellular phosphoproteins, expressed in lymphoid tissues.

## Discussion

Our profiling of APECED autoantibody reactivities yielded a broad autoantibody repertoire and, except type I IFN and Th17 related cytokines, remarkable inter-individual variability. The number of candidate autoantigens ranged to almost thousand reactive proteins. Many of these proteins had lower signal intensities than type I IFN and Th17 cytokines and may partly represent a physiological (i.e., non-pathological) autoimmunity in the patients, similarly to natural autoantibodies in healthy individuals. However, in contrast to the genes activated by Aire in mTECs, only the minority was tissue-restricted proteins as we found many of the targets expressed in cell types with shared presence in lymphoid organs. Our study is in contrast to previous report (15), which identified only two novel targets (PDILT and MAGE-B2), also detected by our screening here, and with a key observation that autoimmunity in APECED preferentially targets molecules with restricted tissue expression profiles. The discrepancy may be related to conditions of serum samples, serum dilutions, differences in the versions of the Protoarray platforms, screening process and data analysis. While the previous study set highly stringent criteria considering only antigens that were common to the patient group we included the rare patient-specific reactivities, which to some extent may contain false positives.

Among tissue-restricted proteins, we found reactivity to many CT-As, implying a central role of the thymus in antitumor immunity. These included members of MAGE-A, MAGE-B, and GAGE families, and in addition sperm-specific proteins, recognized by several (including female) patient sera. By contrast, we did not detect reactivities to MAGE-D or MAGE-E, the non-CT-A members of MAGE protein family (43) that are expressed in all tissues. Importantly, the only cell type expressing CT-As outside of tumors and testis is mTEC (44), and in agreement with our findings of extensive anti-CT-A immunity in APECED, Aire-deficient mice confer strong rejection of melanoma by thymus-dependent T cell responses (45–47). Furthermore, Aire has a dual role in the maintenance of immune tolerance as it also drives the development of a subset of regulatory T cells; indeed, suppressive Aire-dependent regulatory T cells were recruited to tumor sites in a mouse model of prostate cancer (48). Thus, our data in human patients highlight the role of AIRE in modulating immune responses to CT-As with implications for cancer immunotherapy.

The repertoire and properties of target autoantigens, which represent the collection of proteins without any recognizable rule, have been enigmatic. Clearly, the property to become an autoantigen is not a single intrinsic feature but a variable combination of protein characteristics. These features may include antigen structure, susceptibility to proteolytic cleavage, localization in apoptotic blebs, and release into the extracellular space (49). Other studies have highlighted structural motifs (50), evolutional conservation (41), posttranslational modifications (39), and a pro-inflammatory milieu of tissues (51). Post-translational modifications have been associated with various autoimmune diseases and include citrullination in rheumatoid arthritis (52), deamidation in celiac disease (53) and phosphorylation in systemic lupus erythematosus (54). We indeed found enrichment of evolutionarily conserved intracellular phosphoproteins, suggesting this posttranslational modification as one key factor in eliciting autoantibodies, and that specific protein properties contribute to B cell autoimmunity. In parallel to B cell epitopes, antigen phosphorylation is widespread and preserved among T cell epitopes on major histocompatibility complex (MHC) class I and II (55, 56), and deregulated phosphorylation creates tumor-specific neoantigens by affecting the antigenic identity or binding to MHC (57).

The high and neutralizing autoantibody titers to pro-inflammatory cytokines type I IFNs, IL-17A, IL-17F, and IL-22 implicate the inflammatory environment in the generation of the APECED autoantigen repertoire, although their scarcity in Aire-deficient mice (29) remains unexplained. Second, as high titer neutralizing anti-IFNα and anti-IFNω autoantibodies are present in AIRE-deficient thymoma and in recently described RAG-hypomorphic patients who lack AIRE expression (58), this strongly suggests the impairment of AIRE-dependent thymic tolerance in the development of anti-cytokine antibodies in humans. However, type I IFNs and Th17 cytokines are not restricted in their expression to mTECs; likewise the expression of GAD1, steroidogenic enzymes CYP17A1 and CYP21A2, and thyroglobulin is present in other thymic cells and they have no correlation with AIRE expression (59–61), in contrast to insulin and the alpha-subunit of AChR (59). In AIRE-deficient thymoma samples, some APECED autoantigens are expectedly under-expressed, but many are not, including several adrenocortical, gonadal and neuro-ectodermal targets (60, 62). Hence, other cell types in the thymus should contribute to the negative selection of autoreactive T cells. Thus, other mechanisms should be considered, in addition to the role of AIRE in shaping negative selection by upregulating the expression of TR antigens. Indeed, the very early onset of clinical symptoms in APECED patients have suggested that AIRE deficiency may create an actively immunizing tissue environment where tolerance of AIRE-independent antigens is broken (63).

Despite the fact that approximately 90% of the overall group of autoantigens were not tissue-restricted, the correlation of TR-specific subset with mature mTEC population and Aire dependency was strikingly strong. Thus, our results demonstrate two distinct subgroups of autoantigens; first, the smaller subgroup, which consists of approximately 10% of autoantigens, is expressed in specific tissues, and of which many are expressed in mTECs under Aire regulation. The breakdown of tolerance to these APECED autoantigens is likely driven by their lack of expression in the thymus, causing the defect in negative selection of autoreactive thymocytes. The characteristic members of this group are GIF, CYP2A7, LCN1, as well as GAD2, the expression of which followed AIRE’s expression pattern in thymomas (62), and conceivably CT-As, for which AIRE-dependency in human thymus remains unknown. By contrast, the second subgroup of NTR autoantigens is associated with intracellular phosphoproteins, expressed in multiple tissues with enrichment in lymphoid tissues (lymph node, spleen, bone marrow, and tonsils), and as such represents the mTEC-independent breach of tolerance, albeit caused by AIRE mutations. Autoantibodies to these proteins emerge by actions of yet unknown mechanisms associated, for example, with apoptotic cell death or by dysfunctional B cell tolerance during differentiation in lymphoid tissues. Our findings are supported by RNA-seq analysis of autoantigen-encoding genes, which identified a subset of autoantigens, associated with autoimmune diseases, to be expressed ubiquitously but enriched in immune tissues (64). It should be noted that, in contrast to other immune tissues, the thymus was not available via Human Protein Atlas (20). Nevertheless, given the proposed hypothesis of active autoimmunization in AIRE-deficient thymus, this raises intriguing questions of whether the second set of lymphoid autoantigens might represent the antigens acquired form apoptotic thymocytes.

Finally, we identified so far unrevealed clinical associations with pernicious anemia, vitiligo, and autoimmune hepatitis. These correlations need further studies as well as the analysis of the expression of target proteins in corresponding diseased organs and tissues. Despite several outstanding correlations of clinical disease and specific autoantibodies in APECED, the majority of autoantibody reactivities seem not to have relevance to specific clinical entities. They can be just a bystander result of T cell responses, not necessarily reflecting full-blown autoimmune attack or their reactivity may depend on other factors, for example, the presence of posttranslational modifications or complexes with nucleic acids, which can operate as adjuvants. In contrast to single autoantigen responses, our results highlight the overall spread of autoantibody repertoire as a driver for the expansion of clinical profiles in APECED. Alternatively, these autoantibodies may have unexpected protective roles as we recently showed a negative correlation of anti-IFNA antibodies with the incidence of T1D (9). The unexpectedly broad APECED autoimmunome forms a unique platform for further analysis of B cell autoimmunity toward self-antigens and their correlation with clinical manifestations.

## Ethics Statement

The study was carried out in accordance with the recommendations of local ethics committees (Finland: HUS Medical ERB, 8/13/03/01/2009; Slovenia: National Medical Ethics Committee number 22/09/09 and 28/02/13; Italy: Ethics Committee Prot. PG/2015/20440; Norway: Research Ethics Committee of Western Norway, health registry number 047.96, bio-bank number 2013-1504, project number 2012/1850; Estonia: Research Ethics Committee of the University of Tartu, 235/M-23) with written informed consent from all subjects. All subjects gave written informed consent in accordance with the Declaration of Helsinki.

## Author Contributions

DF, KKisand, CH, and PP analyzed the Protoarray data. CH and MR assisted and supervised seroreactivity screenings of Protoarrays. MP and ARemm performed LIPS analyses. PA, HP, and JV contributed and supervised bioinformatic analyses of Protoarray data. AP, AM, KP, TB, ØB, AW, EH, NK, KKrohn, and ARanki sampled APECED patient samples and contributed to clinical database. AH and PP supervised research. DF, KKisand, CH, AH, and PP wrote the paper with contributions from other authors.

## Conflict of Interest Statement

The authors CH and MR were the employees of Immunoqure AG when the study was conducted. PP, KKisand, KKrohn, AR, and AH are shareholders of ImmunoQure AG. The remaining authors declare no competing financial interests. The work is relevant US Patent Application US20170051055A1 (Human anti-IFN-alpha antibodies).
