# Supplementary material for: Autoantibody Repertoire in APECED Patients Targets Two Distinct Subgroups of Proteins
Source: Front Immunol. 2017 Aug 16;8:976. doi: 10.3389/fimmu.2017.00976 (PMC5561390; doi:10.3389/fimmu.2017.00976)
Supplement: Supplementary file 1 [file Data_Sheet_1.DOCX]

**Supplementary Table 1**. APECED patients in this study and their characteristics

| Patient characteristics | Number (%) |
| --- | --- |
| Males | 38 (46) |
| Females | 44 (54) |
| Age at the latest sample (mean ± SD) | 31 ± 17 years |
| Mutations |  |
| R139X/R139X | 17 (21) |
| R257X/R257X | 31 (38) |
| Chronic mucocutaneous candidiasis | 78 (95) |
| Hypoparathyroidism | 65 (79) |
| Addison’s disease | 57 (70) |
| Gonadal insufficiency | 24 (29) |
| Type 1 diabetes | 8 (10) |
| Growth hormone deficiency | 12 (15) |
| Hypothyroidism | 19 (23) |
| Pernicious anemia | 9 (11) |
| Autoimmune hepatitis | 11 (13) |
| gastrointestinal dysfunction | 29 (35) |
| Tubulointerstitial nephritis | 6 (7) |
| Keratitis | 22 (27) |
| Vitiligo | 18 (22) |
| Alopecia | 36 (44) |
| Asplenia | 8 (10) |


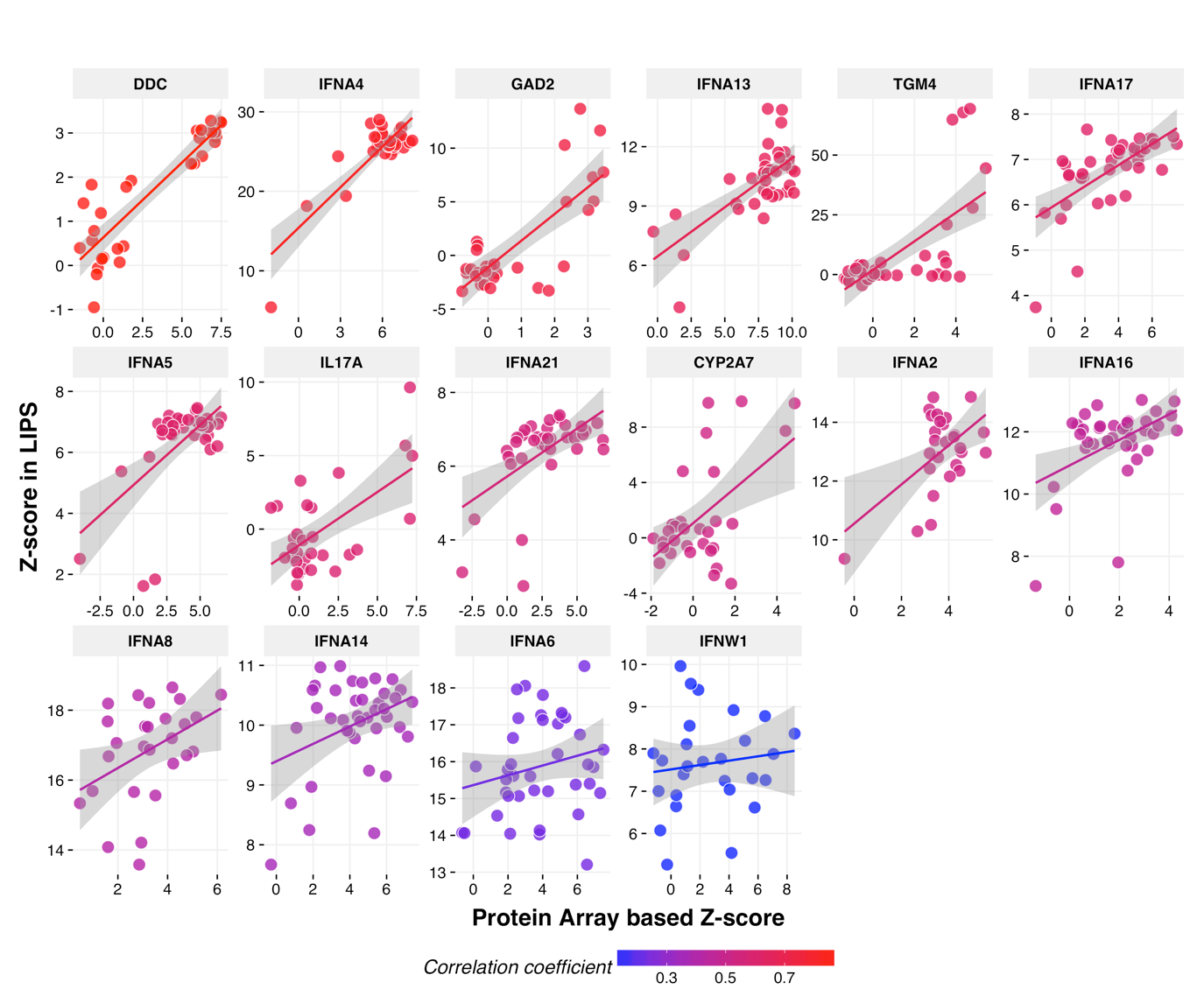


**Supplementary Figure 1.** Correlation analysis of Protoarray and LIPS results in a subset of 30 Finnish APECED patients. Color of dots and fitted linear trends represent the value of correlation coefficient, higher coefficient values are encoded as red color and low or negative correlation as blue.


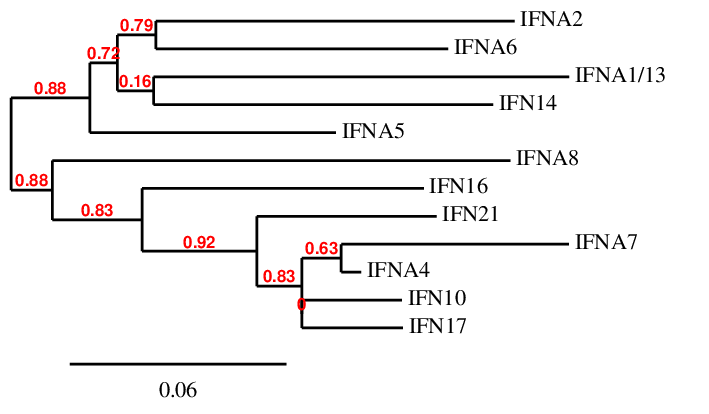


**Supplementary Figure 2.** Phylogenetic analysis of human type 1 IFNA proteins. The human IFNA protein sequences were retrieved from Genbank, aligned and phylogeny was built withPhyML programme and tree rendered with TreeDyn with workflow at <http://www.phylogeny.fr/>.


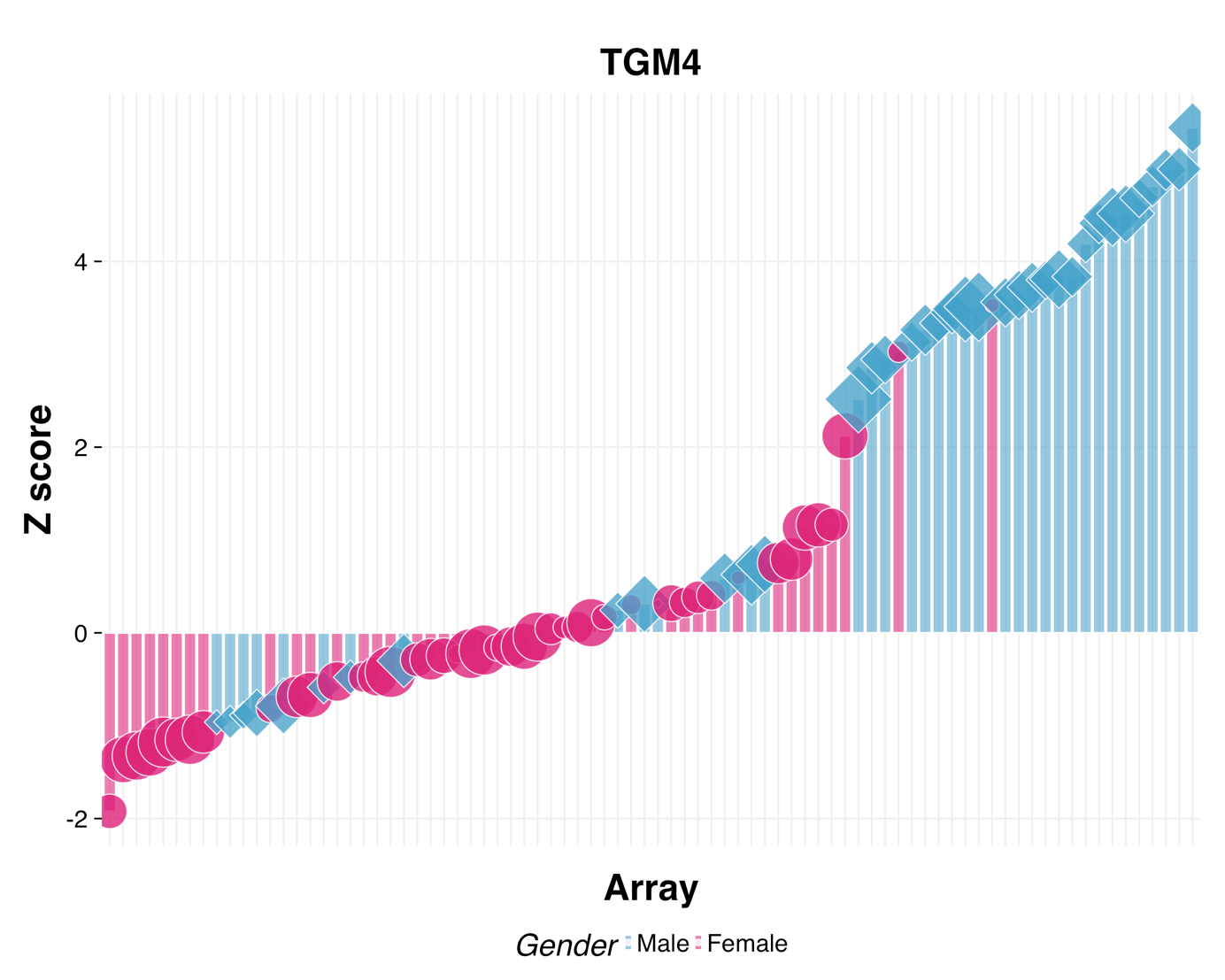


**Supplementary Figure 3.** Gender related difference in TGM4 reactivity. Color represents sex of the patients and the height of the bar indicates the reactivity against TGM4. Size of each diamond (male) or circle (female) is proportional to the age of the patient.


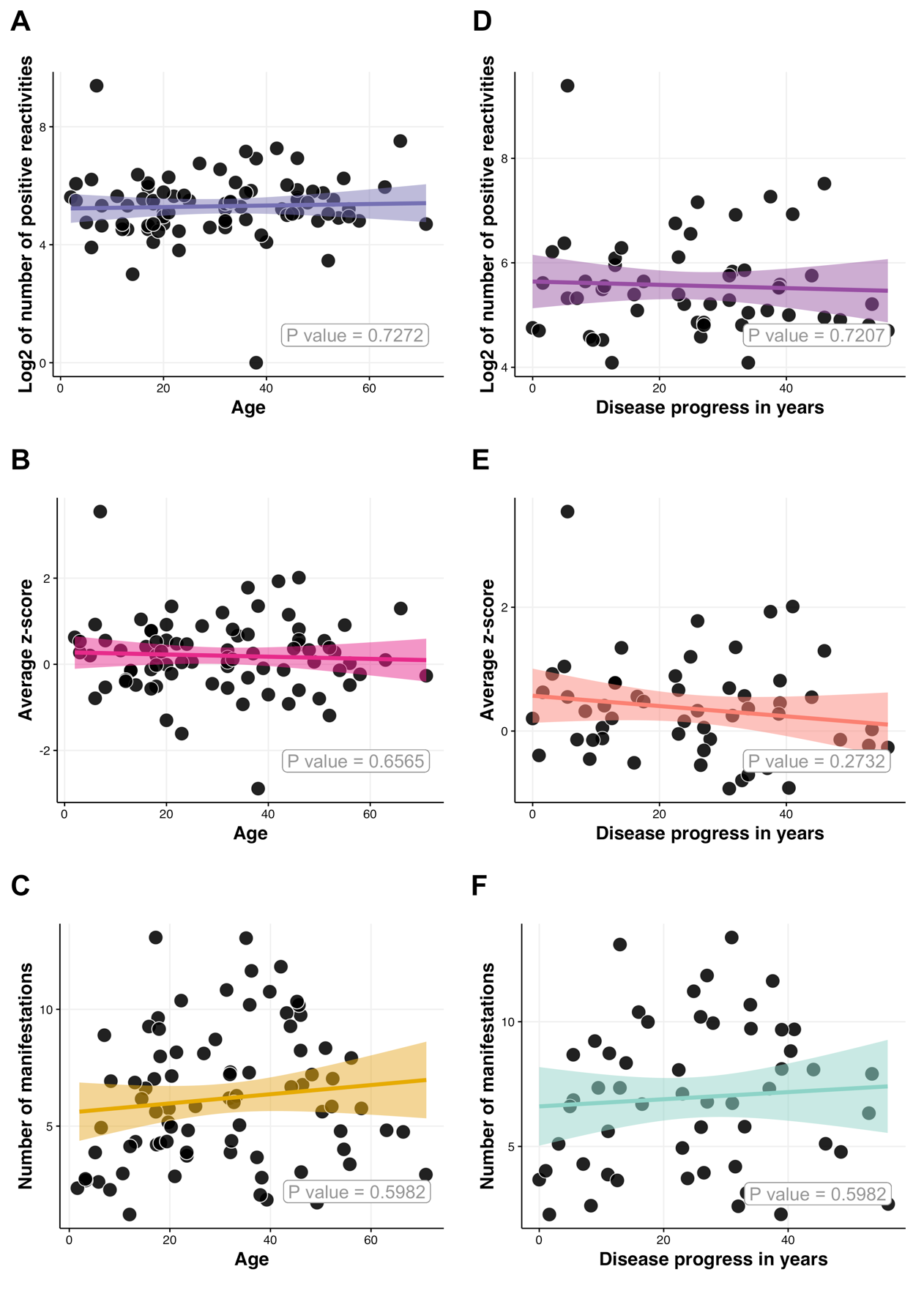


**Supplementary Figure 4**. **A.**Correlation between age of patients and number of autoantigens (logarithmized) with p.value 0.7272. **B.**Correlation between age and average z-score with p.value 0.6565. **C.**Correlation between number of manifestations and age of patients with p.value 0.5982. **D.**Correlation between number of autoantigens (logarithmized) and progress of APECED in years with p.value 0.7207.**E.**Correlation between average z-score and progress of APECED in years with p.value 0.2732. **F.** Correlation between number of manifestations and progress of APECED in years with p.value 0.5982.


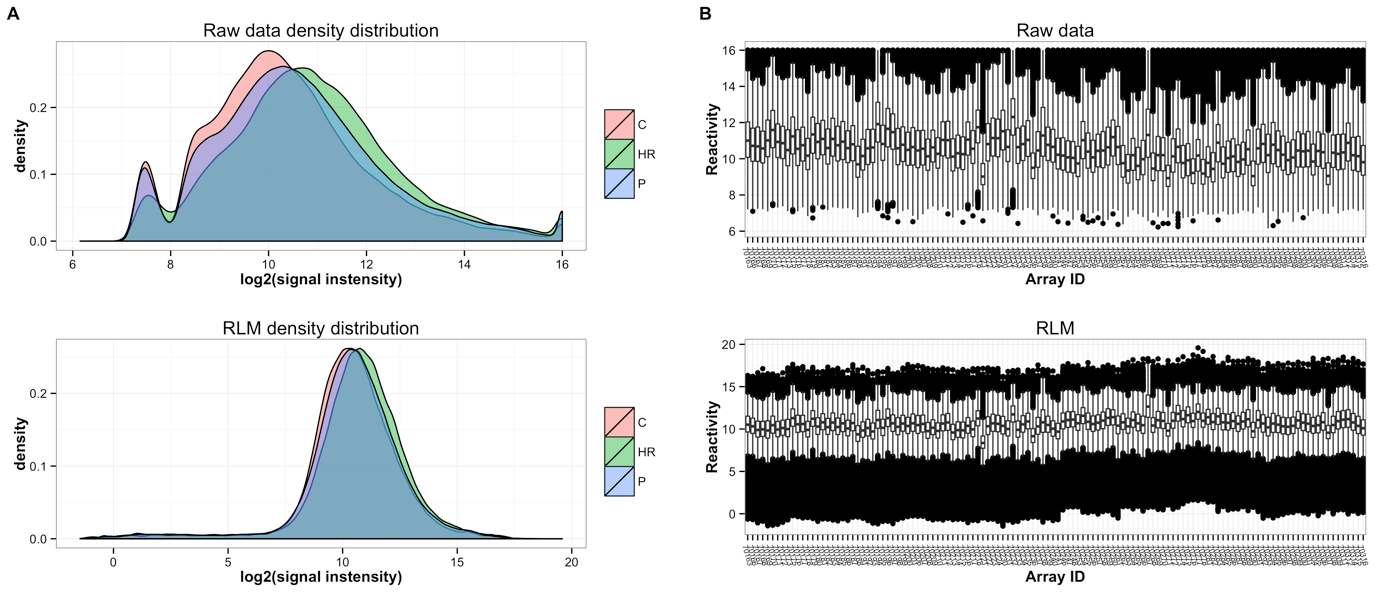


**Supplementary Figure 5**. **A.** Comparison between the distribution of logaritmized protoarray data (above) and RLM normalised (below) signal. Colors represent three meta groups of samples: healthy controls (C), healthy relatives (HR) and patients (P). Normalised signal preserves original inter group variation while transforming signal distribution into normal distribution. **B.** Individual variance of all samples used in the study was visualised. Two panels correspond to raw data (panel above) and data normalised with RLM (panel below). Logarithm was applied to both raw and normalised data for visualisation purposes. We can see that the individual variance for RLM normalised data is reduced comparing to raw data at the same time inter variance between samples is preserved.


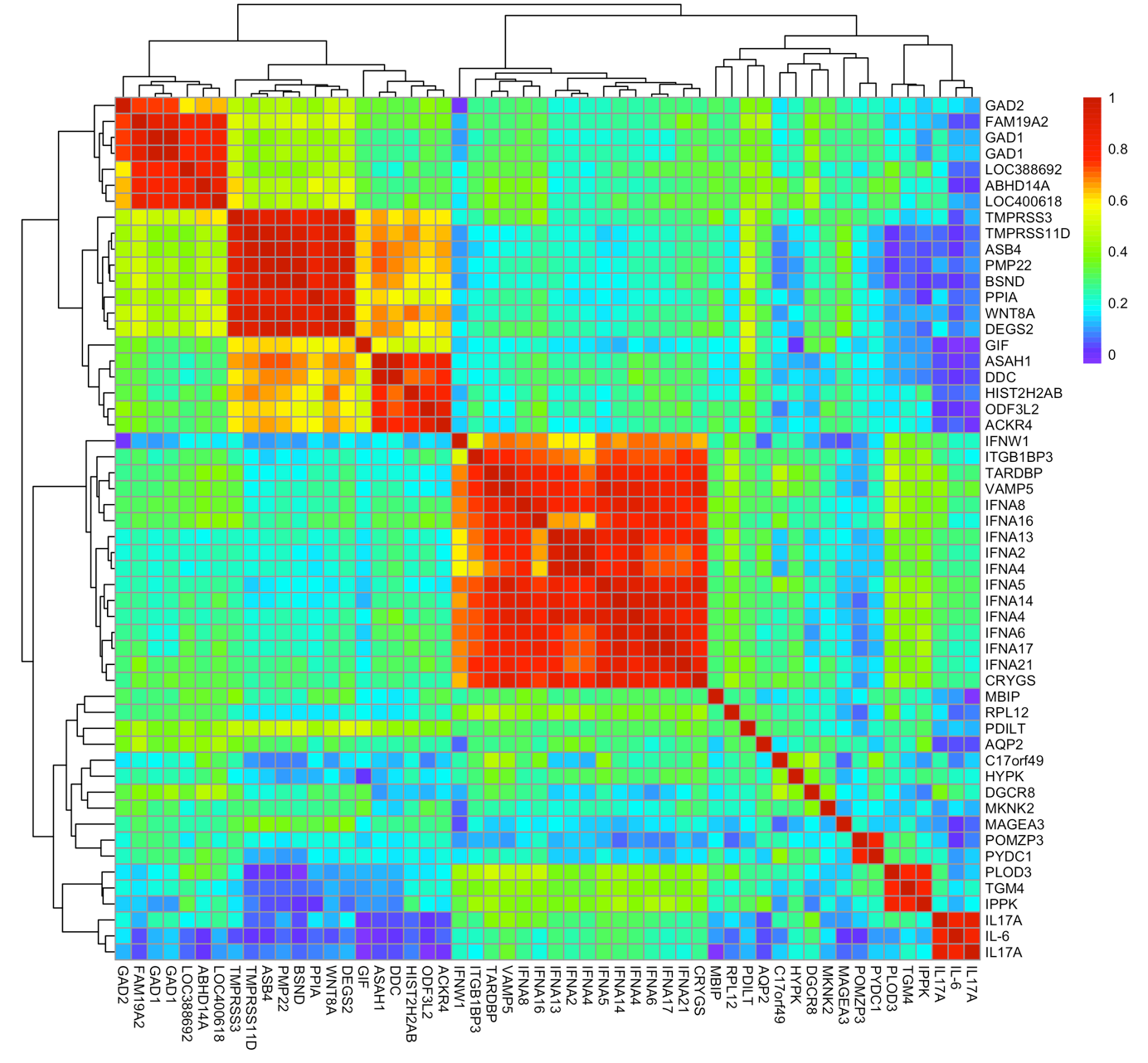


**Supplementary Figure 6**. Heatmap made from correlation matrix of normalized reactivity values with 7 clusters of highly correlated proteins. After the reactivities were normalized, we calculated the z-scores of protein reactivities related to healthy population. We computed a correlation matrix between the most reactive proteins in our analysis. This correlation matrix is represented as a heatmap. Seven clusters of highly correlated proteins could be observed on the heatmap. We hypothesized that proteins that co-occur in the cluster with known autoantibody must have been influenced by these autoantibodies, their signal was skewed. In order to avoid potential technical noise we removed those proteins from our analysis.
